# Adherence to Treatment in Stroke Patients

**DOI:** 10.3390/ijerph16020196

**Published:** 2019-01-11

**Authors:** Emmanouela Cheiloudaki, Evangelos C. Alexopoulos

**Affiliations:** 1 School of Social Sciences, Hellenic Open University, 26335 Patra, Greece; litsachi@hotmail.com; 2 Occupational Health Department, Metropolitan General Hospital, 15562 Athens, Greece

**Keywords:** stroke, recovery, return to work, rehabilitation, compliance, beliefs, Greece

## Abstract

*Background*: Compliance with medication in patients who have suffered stroke is usually not-optimal. This study aims to measure the level of compliance with the treatment and to identify socio-demographic, clinical, and subjective factors related to the long-term compliance of stroke patients with their treatment. *Methods*: 140 patients (66.4% males) suffered an ischemic stroke at least six months old, participated in the survey. Compliance was measured using the Medication Adherence Report Scale and the quality of life by the Stroke Specific Quality of Life questionnaire. Furthermore, the Beliefs about Medicines Questionnaire and the Brief Illness Perception Questionnaire on perceptions about the disease were assessed. The doctor–patient relationship was assessed by the Common-Sense Model of Self-Regulation questionnaire and the family support was assessed by the FSS scale. Univariate and multivariate analysis was employed to identify the significant factors affecting compliance in these stroke patients. *Results*: In 68.6% of patients the compliance was classified as optimal, in 25.7% as partial and as poor in 5.7%; the last two categories were treated as sub-optimal compliance in multivariate analysis. The high compliance was related to patient’s mental state (OR:3.94 95% CI: 1.84–4.46), the perception medication necessity (OR:1.26 95% CI: 1.01–1.56), and the doctor–patient communication (OR:1.76 95% CI: 1.15–2.70). Men showed a lower compliance than women, as well as increased concerns about taking medication (OR: 0.83, 95% CI: 0.69–0.99). Paradoxically, the work /productivity related quality of life was inversely associated with compliance (OR (95% CI): 0.44 (0.23 to 0.82)). *Conclusions*: The perception of medication necessity and the doctor–patient communication are manageable factors associated with compliance in treating patients who have suffered stroke. In addition, rehabilitation and return to work programs should consider these factors when providing support to those persons.

## 1. Introduction

Stroke often leads to death or permanent disability, causing functional or neurological deficits and affects the quality of life of both patients and their families [1,2]. Reduced cognitive function in stroke patients has been associated with reduced ability in daily life. Returning to work and sustaining employment emerge as key goals of rehabilitation and recovery by working-age stroke survivors. Successful return to work after stroke is a major factor in the achievement of high subjective well-being and life satisfaction [3,4].

Stroke related costs are enormous for patients, their families and the society and its rising frequency constitutes a major challenge for health policymakers [5].

The control of the reversible risk factors like diabetes, hyperlipidemia, atrial fibrillation, smoking, and hypertension reduce morbidity and mortality and improve quality of life. Effective tertiary prevention is based on the strict application of the guidelines given by the doctor to the patient which are mainly related to changes in lifestyle and specific medication [6]. However, studies have shown that secondary management of risk factors is not optimal [7]. Compliance with medication constitutes a primary factor of treatment success since suboptimal compliance is a risk factor for secondary stroke or even death [8].

The purpose of the study was to investigate the factors affecting the compliance to treatment of patients who have suffered ischemic stroke. Several factors related to the patient, to his/her beliefs about the disease and its treatment and his/her relationship with the doctor, were investigated.

## 2. Materials and Methods

### 2.1. Study Design

The survey was conducted in the neurology-neurosurgery outpatient clinic of the general hospital of Chania, Crete, during a four-month period (November 2015–February 2016). All patients visited the clinic during this period and fulfilling the eligibility criteria were asked to participate in the study, by giving their informed consent. Eligibility criteria included: (1) Patients who have suffered a first—non-hemorrhagic—stroke at least six months before the interview; (2) patients able to communicate in the Greek language; and (3) patients mentally capable of giving consent and able to comprehend and answer the questionnaire (e.g., patients with dementia and psychiatric disorders were excluded). The research protocol was submitted and approved by both the Hospital Ethics Committee and the Clinic director (Registration number: 16488/8-12-2015), under the conditions of patients’ anonymity and confidentiality and the exclusive use of the study results for research purposes. Participation was voluntary, the stress imposed on patients was limited to a minimum level, while not disrupting the operation of the clinic. Following a brief interview to ensure eligibility and provision of adequate information about the study, the questionnaires were completed by personal interview with patients. From 208 eligible patients 140 agreed to participate (response rate 67.3%) and gave their informed consent. The main reasons for refusals were reservations or fear and previous negative experience on research participation. 

### 2.2. Research Tools

#### 2.2.1. MARS-5 Questionnaire (Medical Adherence Report Scale)

The MARS-5 questionnaire on the adherence to medication, is widely used in stroke patients [9] asks respondents to rate the frequency of performing each of five behavioral aspects of non-compliance, on a five-point scale with the options: always, often, sometimes, rarely, never. Each answer is assigned the values 1 to 5 with: Always = 1, often = 2, sometimes = 3, seldom = 4, and never = 5. Scores are summed up to a scale ranging from 5 to 25, with higher scores indicating higher levels of patient compliance to treatment. In this research compliance under 23 were considered as sub-optimal. However, we have used in a limited extent a trichotomized compliance variable by dividing suboptimal compliance category in two levels: partial compliance (i.e., MARS-5 score 20–23) and poor or no compliance (i.e., MARS-5 score below 23). The Cronbach alpha coefficient was 0.650. 

#### 2.2.2. Stroke Specific Quality of Life Questionnaire (SS-QOL) 

The questionnaire has been extensively used to assess the quality of life in various sub-groups of patients who have suffered stroke and is validated in Greek language [10,11]. The scale consists of 49 questions assess the quality of life of patients with stroke during the last week, including physical, emotional, and social aspects of life. The questions (q) are grouped into 12 sectors and concerns: personal care (SC, 5q), vision (V, 3q), speech (L, 5q), mobility (M, 6q), work/productivity (W, 3q), upper limb function (UE, 5q), mental status (T, 3q), personality (P, 3q), mood (MD, 5q), family participation (FR, 3q), social participation (SR, 5q), and energy (E, 3q). The options in each of the 49 questions depends on the problem level and the rating scale ranges from 1.0 (worst quality of life) to 5.0 (optimum quality of life). The total score is calculated as the average score of the individual sectors and values less than 4.2 are associated with a significant reduction in quality of life after stroke. The Cronbach coefficients were quite high for all subscales and total SSQOL: SSQOL-SC: 0.927, SSQOL-V: 0.851, SSQOL-L: 0.974, SSQOL-SSQOL-M: 0.908, SSQOL-W; 0.935, SSQOL-UE: 0.955, SSQOL-T: 0.827, SSQOL-P: 0.835, SSQOL-MD: 0.774, SSQOL-FR: 0.843, SSQOL-SR: 0.909, SSQOL-E: 0.938, SSQOL-TOTAL: 0.970.

#### 2.2.3. Julkunen Family Support Scale (FSS)

The scale of family support is intended to record the perceived support a person receives from his family and has been validated and used widely in Greece [12,13]. It consists of 13 items (seven are scored inversely) in a Likert scale ranging from 1 (strongly disagree) to 5 (strongly agree). A higher score corresponds to an increased perceived family support. The Cronbach’s alpha was 0.772. Because the scale is intended for people living with or in very close proximity with their family, people who live alone handled as missing values in our analysis. 

#### 2.2.4. Beliefs about Medicines Questionnaire (BMQ) 

The questionnaire assesses cognitive representations of patients about their medication [14] and has been validated in Greek language version [15]. It consists of two parts: (a) a specific part which assesses the beliefs of patients about the medicines prescribed for their personal use, and (b) a general part which assesses beliefs about medicines in general. In this study only, the specific part was used. The specific part consists of two subscales; five questions assess patients’ beliefs on the necessity of prescribed medication and six questions assess their concerns about possible negative effects. Respondents indicate the degree of agreement with each statement in a five-point Likert scale where 1 = strongly disagree and 5 = strongly agree and the rating sum ranges from 5 to 25 for the first subscale and from 6 to 30 for the second one. The higher scores indicating stronger beliefs on the concepts (necessity and concerns) represented by each subscale. The Cronbach’s alpha values were low for the dimension of necessity (0.560) and high for the dimension of concerns (0.779); for the whole specific part was 0.695. 

#### 2.2.5. Brief Illness Perception Questionnaire (BIPQ) 

The BIPQ questionnaire is related to the perceptions of patients about ill-health and is the short version of the Revised Illness Perceptions Questionnaire [16,17]. It is designed to test cognitive and emotional ideas about the disease, describing the process by which individuals respond to a perceived threat to health. 5 questions test the effects of cognitive dimensions of disease (IPQ1- Consequences, IPQ2-Timeline of Disease, IPQ3-Personal Check, IPQ4-Therapeutic Monitoring, IPQ5-Identity) and the remaining three the emotional ones (IPQ6-Concern, IPQ7-Consistency, IPQ8-Emotional Effect). Each question rated from 0 to 10. The total score ranges from 8–80 while questions 3, 4, and 7 are calculated by a reverse scale [16]. An open-ended ninth question asks the respondent to indicate the three most important factors he/she believes has caused his/her illness. This questionnaire has been validated in Greek language [18]. 

#### 2.2.6. CS-SRM Questionnaire

The patient-doctor relationship and communication were tested with the CS-SRM questionnaire which assess whether doctors provide patients with the appropriate information to fully understand their illness and treatment [19]. The questionnaire consists of seven questions refer to specific domain of representations of the disease: causes, identification, timing, control and consequences. The scores are 1 for yes/agree, 0 for no/disagree, and a non-rating option of “do not know” or “not applicable”. The total score ranging from 0–7, with higher scores indicating good communication between doctor and patient [20]. The Cronbach alpha value was 0.561. 

### 2.3. Statistical Methods

The mean and standard deviation were used to describe the continuous variables while the frequency and the % frequency were used for discrete variables. Pearson chi square test was used to test the association between discrete variables and independent samples *t*-test was used for continuous variables comparison between two groups or the one-way ANOVA for more than two groups comparison. Univariate logistic regression was used to assess crude’s Odds ratios and multiple logistic regression was applied to estimate the weighted OR ratios (adjusted OR). OR is related to per point increase of the independent variable scale. In this research, we have used compliance mainly as dichotomized variable but in a limited extent a trichotomized variable was entered a univariate analysis to explore if the trends are constant as a kind of sensitivity analysis. So, optimal compliance is the same both in dichotomized and trichotomized variable (i.e., MARS-5 scores 14 or 25). In dichotomized variable sub-optimal compliance include all scores below 24. In trichotomized variable scores 20–23 were considered as partial compliance while scores below 20 as poor or no compliance. The statistical analysis was done using IBM SPSS Statistics for Windows, Version 21.0 (IBM Corp, Armonk, NY, USA).

## 3. Results

The study sample consisted of 140 patients who have suffered a first ischemic stroke. 93 (66.4%), were male, 16 (11.4%) were living alone and 52 (37.1%) were smokers. The mean age of patients was 64.2 (± 9.0) years old with an age range of 35–79 years (Table 1). Out of the 16 people who living alone, four had no children, 2 had children in other city and 10 in the same city. 52 (37.1%) patients, were current smokers, more among males compared to females (*p* = 0.055). Age and education did not differ significantly between men and women but more women (23.4%) were living alone compared with men (5.4%, *p* = 0.002).

The median number of daily medicines was 4, ranging from 1–12 drugs. In the present study, 73.6% of patients reported no support in taking their medication while 37 patients were supported; mainly by their spouse (70.3%). Females hold mainly the role of the caregiver (88.5%) compared to men (27.3%, *p* < 0.001) (Table 1). The mean compliance score (MARS-5 scale) was 23.7 (± 2.1), the minimum score was 10 and 96 patients (68.6%) were considered as optimally compliant. 

The quality of life total score (SSQOL scale) was on average 4.3 (± 0.7) with a range from 2 to 4.9. The higher mean values were reported for vision related subscale (4.7), self-care (4.7), the upper limb function (4.5) and speech (4.5). The lower values recorded in the social participation (3.9), energy (3.9), and personality (3.4) SS-QOL subscales. The family support total score (FSS scale) ranged from 27–64 with a mean of 59.6 and a median of 61.0. Concerning respondents’ beliefs on the three most important factors causing their illness anxiety, unhappiness and hypertension were reported followed by diabetes, thrombophilia, and lifestyle. The mean score of perceived necessity of medication was 18.8 (range 5–25) and of the perceived concerns 11.1 (range 6–30). In perceptions of respondents about their illness (IPQ) the largest average value was monitored in the therapeutic control scale (8.2 ± 1.7) following by personal control (7.5 ± 1.6) and consistency (7.0 ± 1.6) scales. The perception of the doctor patient relationship as perceived by the patient CS-SMR questionnaire was high (mean 5.9 and a median of 6.0) (Table 2).

In the univariate analysis of the relation of socio-demographic and disease duration on compliance, living status was of importance (OR: 3.27, 95% CI: 1.13 to 9.46). 56.3% of patients living alone did not comply optimally compared to others (28.2%). This is anticipated to partially capture the relation with the marital status. Compliance was also related to disease duration. Compliant patients had significantly less mean illness duration of 3.3 years compared with the non-compliant ones (4.8 years). Gender, age, the number of children, educational level, smoking status, support in taking medication, did not found to be significantly related with compliance (Table 2).

Compliance was related to sight subscale of quality of life related (OR (95% CI): 2.30 (1.20 to 4.43)) and the mental state subscale OoL (OR (95% CI): 2.49 (1.64 to 3.79)) (Table 2). Compliance had a borderline significant with the family support scale (OR (95% CI): 1.07 (0.99–1.16)) and the perceived necessity and concerns scales of medication (BMQ) with ORs (95% CI) of 1.14 (1.01 to 1.30) and 0.89 (0.82 to 0.97), respectively (Table 2). The physician–patient communication (CS-SRM scale) showed also significant relation to compliance (OR (95% CI): 2.11 (1.50–2.96)) (Table 2).

As previously mentioned, we have used compliance as trichotomized variable in a univariate analysis to explore if the trends are constant as a kind of sensitivity analysis (Table 3). In the trichotomized variable sub-optimal compliance was further divided in two groups: the partial compliant group (scores 20–23) and the poor or no compliant group (scores below 20). Partial compliance was reported by 36 people and, poor by 8 individuals. Table 3 shows the comparison of optimal and partial compliance with the poor. Compared with poor compliance partial compliance was related to gender; more male patients reported poor compliance (OR: 0.11, 95% CI: 0.02 to 0.65). Increased concerns related to lower compliance (OR: 0.83, 95% CI: 0.69–0.99) as current smoking also did (OR: 0.15, 95% CI: 0.03–0.84). Living with others were less common among those with poor compliance compared to those with partial and optimal compliance (Table 3). Concerning the quality of life subscales, the single most important was that associated with vision problems with an OR 7.72 for optimal and 5.33 for partial compliance. Other important relations were found between mental status (SSQOL-T OR: 3.31), energy (SSQOL-E OR: 1.90) and mood (SSQOL-MD OR: 2.17) (Table 3). Family and social participation SSQOL subscales exhibited borderline significance while were interrelated with family support (Table 3). All variables exhibited a *p*-value <0.200 were included to multivariate modelling (data not shown in Table). Compared to those with poor compliance, three variable reached statistically significant level for optimal compliance, in multivariate analysis: the medication necessity (OR: 2.18, 95% CI: 1.04 to 4.57); the medication concerns (OR: 0.51, 95% CI: 0.27 to 0.99) and in a borderline level the doctor–patient communication (OR: 3.41, 95% CI: 0.80 to 14.50). As expected, due to the low sample sizes between the poor/no and partial compliance categories, the comparison did not reach statistically significant level in any of the study variables. However, medication necessity, medication concerns and doctor–patient communication showed similar trends (OR: 1.93, 95% CI: 0.93 to 4.02; OR: 0.54, 95% CI: 0.28 to 1.05; and 1.96, 95% CI: 0.45 to 8.45, respectively).

In the final multivariate modeling the dichotomized outcome variable (i.e., MARS-5 scores above 23 as optimal and all the others as sub-optimal compliance) was used. All variables under study on treatment compliance exhibited a *p*-value <0.200 were included to univariate and multivariate modelling (Table 4). The most important relations with compliance were found for mental state (SSQOL-T score) with weighted OR 5.14 (95% CI: 2.64 to 10); good doctor–patient relationship (CS-SRM scale) with weighted OR 1.96 (95% CI: 1.35 to 2.85) and the perceived need for medication with weighted OR 1.29 (95% CI: 1.06 to 1.56) (Table 4). The work /productivity quality of life was inversely associated with compliance (OR (95% CI): 0.44 (0.23 to 0.82)) (Table 4).

## 4. Discussion

In this research, we have studied the compliance to treatment of 140 patients who have suffered ischemic stroke and its relation to various variables. The compliance was measured by the Medication Adherence Report Scale and was treated as dichotomous (optimal or suboptimal) or trichotomous (optimal, partial, and poor) variable. Most patients (68.6%) were categorized as optimally compliant while the 25.7% as partially compliant and the 5.7% as of poor compliance.

The patients’ quality of life score (as measured by the SS-QOL questionnaire) was quite high. These high rates are not uncommon in patients with ischemic stroke and has been associated with high compliance rates, as in our study [21]. Compliance was related to less visual and mental problems which have been found to affect compliance [22]. Cognitive disorders have been shown to adversely affect treatment compliance [9,23] as well as emotional disorders such as anger, fatigue and stress, confirming the results of this research. 

In our study, the age of the patients was not related with compliance, although in another study, younger stroke patients reported that they often forgot some of their doses [9]. Other studies also support higher compliance rates in elderly patients [24,25,26].

We found that male patients showed a lower compliance than women, who hold significantly more often the caring role compared to men. This finding is consistent with previous research in which most non-compliant patients were men and often were depended in their wife’s help or relatives’ support [27]. 

Living alone was related to lower compliance in our study, as other studies have also shown [23,27]. Lack of family support and the feeling of conflict within the family have also found to decrease compliance in patient treatment [2,28].

The inverse relation of compliance with time (disease duration) found in our study has been confirmed in several studies which consist a severe medical care problem in chronic disease management [27,29,30,31,32].

The compliant patients reported more positive beliefs about medicines, deemed them as necessary ("necessity") and less negative beliefs questioning the need and the benefits or worrying about the damage they might cause ("concerns"), as measured by the BMQ questionnaire. Similar results were observed in a study from the United Kingdom, in which statistically significant correlation found between anxiety and reduced compliance [9]. In other studies, non-compliant patients reported lower scores on the need and benefits and higher ones on concerns [27,33].

We did not find a significant relation between the perception of the disease (as measured by IPQ questionnaire) and compliance. Several studies have also found stronger correlations between medication beliefs than disease perceptions [34,35]. This is also consistent with the extended model of Leventhal (2011), according to which perceptions of the disease is directly related to compliance but the beliefs about medication are often stronger [36].

The relation of compliance with the doctor–patient communication is well established. Numerous studies have emphasized that the effective communication between patient and doctor as well as the reduction of any maladaptive belief that patients have about health or illness is of large importance in patient compliance [18,37,38,39,40,41]. Moreover, the need to optimize care under the increasing pressures of ageing, multi-morbidity, disease chronicity is constantly increasing. Within a primary healthcare setting the implementation of chronic care model interventions improved outcomes and patient compliance with treatment. However, their implementation is restricted by various factors. Considerable resources are needed to support implementation and sustainability and human factors played essential roles since both healthcare providers should be prepared for the implementation, and patients should be supported to receive care changes [42]. In this context and under the initiative of people-centered and integrated care, the role of care manager (i.e., specially trained nurses) seem effective to empower patients to make lifestyle changes and to achieve better compliance to treatment and care recommendations [43].

A noteworthy finding in our study was that those patients reported better quality of life in the domain of work and productivity and in a lesser extent also to mobility were far less compliant. This underlies the need to focus on these patients when returning to work and to monitor them during the whole recovery process. So, in addition to the doctor–patient relationship the occupational health services, where available, should continuously assesses the progress and medication adherence of these vulnerable productive people.

The specific health care system and study setting may influence generalizability of our findings. The use of self-report measures for all variables under study is a limitation of our research design; however, is very common in this type of research in which is difficult to utilize additional resources in an outpatient setting. The main limitation is the low sample size given the high number of variables collected. We have treated this weakness by applying a *p*-value limit from univariate logistic analysis (*p* < 0.200) in a backward logistic regression modelling instead of a complete logistic regression model. Further research should consider these limitations in order to increase the utility of findings and application to practice.

## 5. Conclusions

According to our results adherence to treatment in patients with stroke associated with a variety of social, medical, and personal factors. The disease perceived severity, the doctor–patient relationship and the patient’s perception of medication need were all related to compliance. Interventions aimed at compliance are complex and include self-monitoring schemes, counseling and supportive care. In a patient-centered and integrated care, personal beliefs of patients should be carefully considered, and healthcare providers need to recognize the concerns patients have for medicines, stressing the need of these benefits, making the shape simple and adjusted to the lifestyle of the patient, with a view to the reduction of a secondary stroke episode.

## Figures and Tables

**Table 1:** Patients characteristics.

Study Variable	Males (*n* = 93)		Females (*n* = 47)		All (*n* = 140)		
	Mean	SD	Mean	SD	Mean	SD	*p*
Age (years)	65.1	8.5	62.3	9.7	64.2	9.0	0.088
Number of children	2.3	1.9	2.1	1.0	2.3	1.1	0.222
	*n*	%	*n*	%	*n*	%	
Living status							
Living alone	5	5.4	11	23.4	16	11.4	0.002
Living with others	88	94.6	36	76.6	124	88.6	
Children residence							
Same house	15	17.4	4	9.3	19	14.7	0.436
Same city	64	74.4	36	83.7	100	77.5	
Another city	7	8.1	3	7.0	10	7.8	
Education							
Primary	37	39.8	23	48.9	60	42.9	0.388
Secondary	36	38.7	18	38.3	54	38.6	
Higher	20	21	6	12.8	26	18.6	
Smoker							
Current	38	40	14	29.8	52	37.1	0.055
Never	30	32	25	53.2	55	39.3	
Ex-	25	26	8	17.0	33	23.6	
Support in medication							
Never	67	72	36	76.6	103	73.6	0.564
Always/sometimes	26	28	11	23.4	37	26.4	

**Table 2:** Study variables and its relationship with compliance.

Study Variable	All		Patients Compliance				
			Suboptimal		Optimal		
			*n*	%	*n*	%	*p*
Gender (m/f)	93/47		29/15	31.2/31.9	64/32	68.8/68.1	NS
Living with others/alone	124/16		35/9	28.2/56.3	89/7	71.8/43.8	**0.023**
Educational level (higher/lower)	26/114		5/39	19.2/34.2	21/75	80.8/65.8	NS *
Current smoker (no/yes)	88/52		27/17	30.7/32.7	61/35	69.3/67.3	NS
	Mean	SD	Mean	SD	Mean	SD	
Age (y)	64.2	9.0	63.8	10.1	64.3	8.5	NS
Disease duration (y)			4.8	5.2	3.3	3.2	**0.048**
SSQOL							
Self-care (SC)	4.7	0.7	4.8	0.5	4.6	0.7	NS
Vision (V)	4.7	0.6	4.5	0.8	4.8	0.4	**0.012**
Language (L)	4.5	0.9	4.5	0.8	4.5	0.9	NS
Mobility (M)	4.2	0.9	4.4	0.6	4.1	1.0	0.125
Work/Productivity (W)	4.1	1.0	4.3	0.7	4.0	1.1	0.111
Upper Extremity function (UE)	4.5	0.9	4.7	0.6	4.5	0.9	NS
Thinking (T)	4.2	1.0	3.6	1.1	4.4	0.8	**<0.001**
Personality (P)	3.4	1.1	3.2	1.2	3.5	1.1	0.169
Mood (MD)	4.3	0.7	4.2	0.9	4.4	0.7	0.164
Family roles (FR)	4.4	0.9	4.3	1.1	4.5	0.9	NS
Social roles (SR)	3.9	1.0	3.8	1.0	4.0	1.0	NS
Energy (E)	3.9	1.2	3.6	1.4	4.0	1.1	0.119
All subscales	4.3	0.7	4.2	0.7	4.3	0.7	NS
FSS	59.6	5.1	58.2	8.0	60.2	3.2	0.080
BMQ Necessity	18.8	2.8	18.0	3.7	19.1	2.3	**0.038**
BMQ Concern	11.1	4.3	12.6	4.6	10.4	3.9	**0.004**
IPQ	39.0	11.7	39.8	10.7	38.6	12.2	NS
CS-CRM	5.9	1.3	5.07	1.48	6.29	1.08	**<0.001**

In bold: *p* < 0.05; NS: *p* > 0.20; * educational level had three subcategories.

**Table 3:** Univariate analysis of factors affecting the level of compliance (optimal or partial vs. poor/no) of stroke patients.

Study Variable	Adherence					
	Optimal			Partial		
	OR	95%LL	95%UL	OR	95%LL	95%UL
Age (years)	1.01	0.97	1.05	1.00	0.92	1.08
Gender (RC = females)	1.00	0.92	1.09	**0.11**	**0.02**	**0.65**
Living with others	**12.71**	**2.61**	**62.05**	**6.20**	**1.16**	**33.17**
Number of children	0.80	0.47	1.78	1.41	0.63	3.14
Education (RC = Higher)						
Primary-secondary	1.54	0.34	6.90	1.56	0.32	7.70
Current Smoker	**0.19**	**0.04**	**1.00**	**0.15**	**0.03**	**0.84**
Support in medication	0.33	0.08	1.44	0.33	0.07	1.62
SSQOL						
Self-care (SC)	1.21	0.51	2.91	2.40	0.72	7.99
Vision (V)	**7.72**	**2.82**	**21.16**	**5.33**	**1.86**	**15.3**
Language (L)	1.24	0.62	2.48	1.38	0.64	2.97
Mobility (M)	0.97	0.43	2.15	1.56	0.60	3.86
Work/Productivity (W)	1.08	0.56	2.11	1.69	0.78	3.67
Upper Extremity function (UE)	1.07	0.50	2.32	1.55	0.64	3.77
Thinking (T)	**3.31**	**1.63**	**6.74**	1.41	0.71	2.81
Personality (P)	1.89	0.93	3.85	1.64	0.77	3.45
Mood (MD)	**2.17**	**1.02**	**4.64**	1.78	0.79	4.00
Family roles (FR)	1.78	0.97	3.28	1.72	0.88	3.37
Social roles (SR)	1.80	0.98	3.32	1.76	0.91	3.41
Energy (E)	**1.90**	**1.06**	**3.39**	1.65	0.90	3.05
All subscales	2.18	0.92	5.16	2.31	0.89	6.05
FSS	**1.13**	**1.01**	**1.26**	1.06	0.96	1.18
BMQ Necessity	**1.32**	**1.05**	**1.68**	1.20	0.95	1.53
BMQ Concern	**0.76**	**0.64**	**0.90**	**0.83**	**0.69**	**0.99**
IPQ Total	0.98	0.92	1.04	0.98	0.92	1.05
CS-SRM	**2.57**	**1.56**	**4.32**	1.27	0.82	1.97

RC = Reference Category; in bold: *p* < 0.05; in shading: variables with *p* < 0.05 in multivariate multinomial analysis.

**Table 4:** Multivariate modelling of study variables on treatment compliance (optimal vs. all other) of stroke patients.

Study Variable	Full Model *				Final Model *			
	OR	95% LL	95% UL	*p*	OR	95% LL	95% UL	*p*
Living with others	2.11	0.24	18.42	NS				
Disease duration (y)	0.88	0.76	1.03	0.115				
SSQOL								
Vision (V)	2.30	0.66	8.04	0.191				
Mobility (M)	0.46	0.11	1.98	NS				
Work/Productivity (W)	0.45	0.15	1.33	0.150	**0.44**	**0.23**	**0.82**	**0.010**
Thinking (T)	**3.94**	**1.84**	**8.46**	**<0.001**	**5.14**	**2.64**	**10.00**	**<0.001**
Personality (P)	0.89	0.54	1.46	NS				
Mood (MD)	0.75	0.22	2.54	NS				
Energy (E)	1.35	0.60	3.02	NS				
BMQ Necessity	**1.26**	**1.01**	**1.56**	**0.038**	**1.29**	**1.06**	**1.56**	**0.010**
BMQ Concern	0.90	0.78	1.04	0.156				
CS-CRM	**1.76**	**1.15**	**2.70**	**0.009**	**1.96**	**1.35**	**2.85**	**<0.001**

* Full model: all variables with *p* < 0.2 in univariate analysis; Final model: variables retained in the final model by forward selection; in bold: *p* < 0.05; NS: *p* > 0.20.
